# Consistent Hand Dynamics Are Achieved by Controlling Variabilities Among Joint Movements During Fastball Pitching

**DOI:** 10.3389/fspor.2020.579377

**Published:** 2020-11-17

**Authors:** Tomoyuki Matsuo, Tsutomu Jinji, Daisaku Hirayama, Daiki Nasu, Yoichi Katsumata, Yoshitaka Morishita

**Affiliations:** ^1^Department of Health and Sport Sciences, Graduate School of Medicine, Osaka University, Toyonaka, Japan; ^2^Faculty of Human Development, Kokugakuin University, Tokyo, Japan; ^3^Sports R&D Core, University of Tsukuba, Tsukuba, Japan; ^4^Sports Brain Science Project, NTT Communication Science Laboratories, Nippon Telegraph, and Telephone Corporation, Atsugi, Japan; ^5^Faculty of Applied Biosciences, Tokyo University of Agriculture, Tokyo, Japan; ^6^Department of Sport and Health Science, Ritsumeikan University, Kusatsu, Japan

**Keywords:** movement variability, covariation, redundancy, direct kinematics, randomization, baseball

## Abstract

This study aimed to determine whether covariations among joint movements are utilized to stabilize hand orientation and movement and to determine which of the upper or lower extremities make effective use of the covariation. Joint angles during pitching were measured in 12 skilled baseball pitchers, using a motion capture system. The joint angles in 10 successful trials were used for the reconstructed motions. The reconstructed motion in the first condition was the same as for the measured motion. In the second condition, the reconstructed motion was generated with joint angles that were pseudo-randomly selected to artificially break off covariation in the measured joint-angle combination. In the third and fourth conditions, the reconstructed motions were generated with the same joint-angle combinations as the measured angles in the throwing arm and the stride leg, respectively, but pseudo-randomly selected in the other joint angles. Ten reconstructed motions were generated for each condition. Standard deviations (SDs) of hand orientation and movement direction were calculated and compared among the conditions. All SDs for the first condition were the smallest among the conditions, indicating that the movements in the measured condition used the covariation in joint angles to make the hand movement stable. The results also illustrated that some SDs in the fourth condition were smaller than those in the third condition, suggesting that the lower extremity made effective use of the covariation. These results imply that it is necessary not only to reduce variability in each joint but also to regulate joint movements to stabilize hand orientation and movement.

## Introduction

It is well-known that skilled performers can adjust their movements to be more consistent from one trial to the next. Professional baseball pitchers demonstrate smaller variability in several kinematic parameters than youth and high school baseball pitchers (Fleisig et al., 2009). Consistent movement with good mechanics is recommended and purportedly contributes to pitching success (Meyers and Gola, 2000). According to a study involving nine college baseball pitchers, the mean standard deviations (SDs) (±SD) of the release location were 5.7 ± 2.2 and 3.8 ± 1.5 cm in the horizontal and vertical axes, respectively, despite the fact that four different types of pitches had been thrown (Whiteside et al., 2016). The SDs were within the diameter of a standard baseball (7.3 cm).

The aforementioned results suggest that consistent movement by a pitcher plays an important role in maintaining accuracy during pitching. This strategy is referred to as stochastic noise reduction, which is one of the strategies employed in reducing inter-trial variability (Müller and Sternad, 2004).

It is similarly well-known that humans always demonstrate inter-trial fluctuations in movements (Bernstein, 1984). Since the human body has many mechanical degrees of freedom (DoFs), inter-trial fluctuations continuously arise in any DoF from various sources due to inherent neurological or physiological noises (Cusumano and Dingwell, 2013). However, humans can achieve a required task employing a different strategy by using abundant DoFs (Côté, 2014). To maintain the motion of the end effector invariant despite fatigue, more proximal segments were employed to compensate for changes when fatigue occurred in a local joint movement (Côté et al., 2002).

These facts prompted the question of how the hand moves consistently to throw a baseball accurately even though there are so many DoFs of joint movements in the whole body. These DoFs may influence throwing performance and the tendency to incur some injuries.

The permutation (randomization) method was used to resolve the above question (Müller and Sternad, 2003). In this method, it is assumed that a skillful movement uses the covariation among joint angles to make an end effector movement stable. A skillful movement is achieved by controlling the DoFs, and creating covariations is one way of controlling them (Turvey et al., 1982). The covariation among joint angles is removed by a randomization procedure. Then the variabilities generated by the original data and the covariation-free data are compared.

If the stochastic noise reduction strategy is solely adopted, then the inter-trial fluctuations of the result variables measured actually remain in the range of the variabilities generated by the covariation-free motion. Here, for baseball pitching, the result variable is the orientation and/or movement direction of the throwing hand at the instant of ball release, and the execution variable means a joint movement. Conversely, if the covariation strategy is used, then the variability of the measured result variable can be lower than the lower limitation of the variabilities generated by the covariation-free motion, that is, the variability generated in an execution variable is canceled out by the other execution variable(s). Then variability of the actually measured result variable can be lower than that expected under the covariation-free condition.

The covariation among joint angles was observed in treadmill walking to stabilize the position of center of mass and step parameters (Verrel et al., 2010). Although the adopted method is different, the covariations among joint angles were observed to be in control of the hand path velocity and movement direction in Frisbee throwing while restricting trunk movement (Yang and Scholz, 2005) and in the orientation of the clubhead during a driver shot in golf (Morrison et al., 2016).

To achieve high consistency in the result variable, it appears that, in addition to the stochastic noise reduction strategy, the covariation strategy may also be utilized in baseball pitching. To the best of our knowledge, which strategies are employed has yet to be clarified. Therefore, the first purpose of this study was to elucidate the utilization of the covariation strategy among joint movements in stabilizing hand movement. It was hypothesized that the covariation strategy could be used in stabilizing the hand orientation and movement direction at the instant of ball release.

The second purpose of this study was to investigate which of the covariations in the upper or lower extremities reduces the variabilities of the hand orientation and movement direction more effectively at the instant of ball release. Recently, it was reported that the forearm of the throwing arm is supinated for a short period at the instant of ball release, followed by drastic pronation, to meet the need of the task requirement (Matsuo et al., 2016). The supination correlated with the shoulder internal rotation and horizontal adduction, suggesting that certain covariations were associated with movements in the throwing arm motion.

On the contrary, some baseball coaches indicate that a stable body position generated by a stable pivot leg and a stable stride leg is one of the most important factors responsible for accurately throwing a baseball (Meyers and Gola, 2000). This indicates that the covariation may be associated with joint movements in the legs. Thus, it was hypothesized that the effect of the covariation on the variability of the hand orientation and movement direction was more significant in the lower extremity since the distance of lever arm from the lower extremity to the hand is much longer than that from elbow and shoulder joints. A small change in the lower extremity might result in a much larger change in the hand orientation and movement direction than that of the upper extremity.

Thus, it was hypothesized that a covariation existed in both the upper and the lower extremities and that the effect on the variability of the result variables was more significant in the lower extremity since the distance of lever arm from the lower extremity to the hand is much longer than that from elbow and shoulder joints.

## Materials and Methods

### Participants

After a pilot study with nine participants, a priori power analyses on all dependent variables explained below (α = 0.05, β = 0.20, effect size = 0.71) were performed to determine the minimum sample size. Then a large effect was determined so that half of the variation could be explained. Twelve male semi-professional overhand and three-quarter hand pitchers participated in this study. Ten of the participants were right-handed, and two were left-handed. The mean height, weight, and age (±SD) were 1.80 ± 0.04 m, 73.9 ± 5.1 kg, and 22.5 ± 2.0 years, respectively. A standard baseball (mean ball mass was 0.145 kg) was thrown by the participants. In 10 successful trials, the mean speed and mean deviation from the target were 37.0 ± 0.9 m/s and 0.14 ± 0.03 m, respectively. The successful trials will be explained below. All participants were competitive and had experienced no throwing injuries requiring surgery for at least the previous 2 years. The study was conducted in accordance with the World Medical Association Declaration of Helsinki code of ethics for experiments involving humans and was approved by the Institutional Research Ethics Committee at Osaka University. Each participant provided written informed consent prior to participation.

### Procedure and Apparatus

The participants prepared just as if they were going to pitch in an actual game, including pitching practice. After the warm-up, they had 50 retro-reflective markers placed on body landmarks and four markers on the ball (Supplementary Table 1). After attaching the markers, the participants were instructed to warm up for a second time, including pitching on the indoor mound. All participants declared that none of the markers affected their movement and performance.

After pitching practice, participants threw 15 fastballs at the target with a concentric circle, which was located at the simulated position of the catcher's mitt (19.9 m from the pitching rubber). After several minutes of rest, another set of 15 fastballs was thrown. The fastest 10 pitches that hit the target (diameter of 0.29 m) were selected for subsequent analyses as “successful trials” (Supplementary Table 2). For three participants, an additional 5–14 pitches were performed because the number of pitches qualitatively judged to hit the target on the spot failed to satisfy the criteria or because some pitches failed to obtain sufficient motion capture data.

During data collection, each marker position was obtained using a 16-camera VICON MX motion analysis system (sampling frequency: 1 kHz) and NEXUS software (Vicon, Oxford, UK). The ball speed was measured using a radar gun (PSK-DSP, Mizuno, Tokyo, Japan) located behind the target. The ball location was obtained using a digital camcorder (GZ-MG40, JVC, Yokohama, Japan) and Frame-DIAS V software (DKH, Tokyo, Japan).

### Data Processing and Analysis

A fourth-order low-pass digital Butterworth filter was used for data smoothing. The cut-off frequency was determined in the residual analysis (81.8 ± 5.1 Hz) (Winter, 2005).

The instant of ball release was determined from the drastic increase in the derivative of the distance between the marker on the third fingertip and the center of the ball. The center of the ball was calculated (using the non-linear least-squares method) from the four markers on the ball and the radius of the ball (Matsuo et al., 2018).

**Figure 1:**
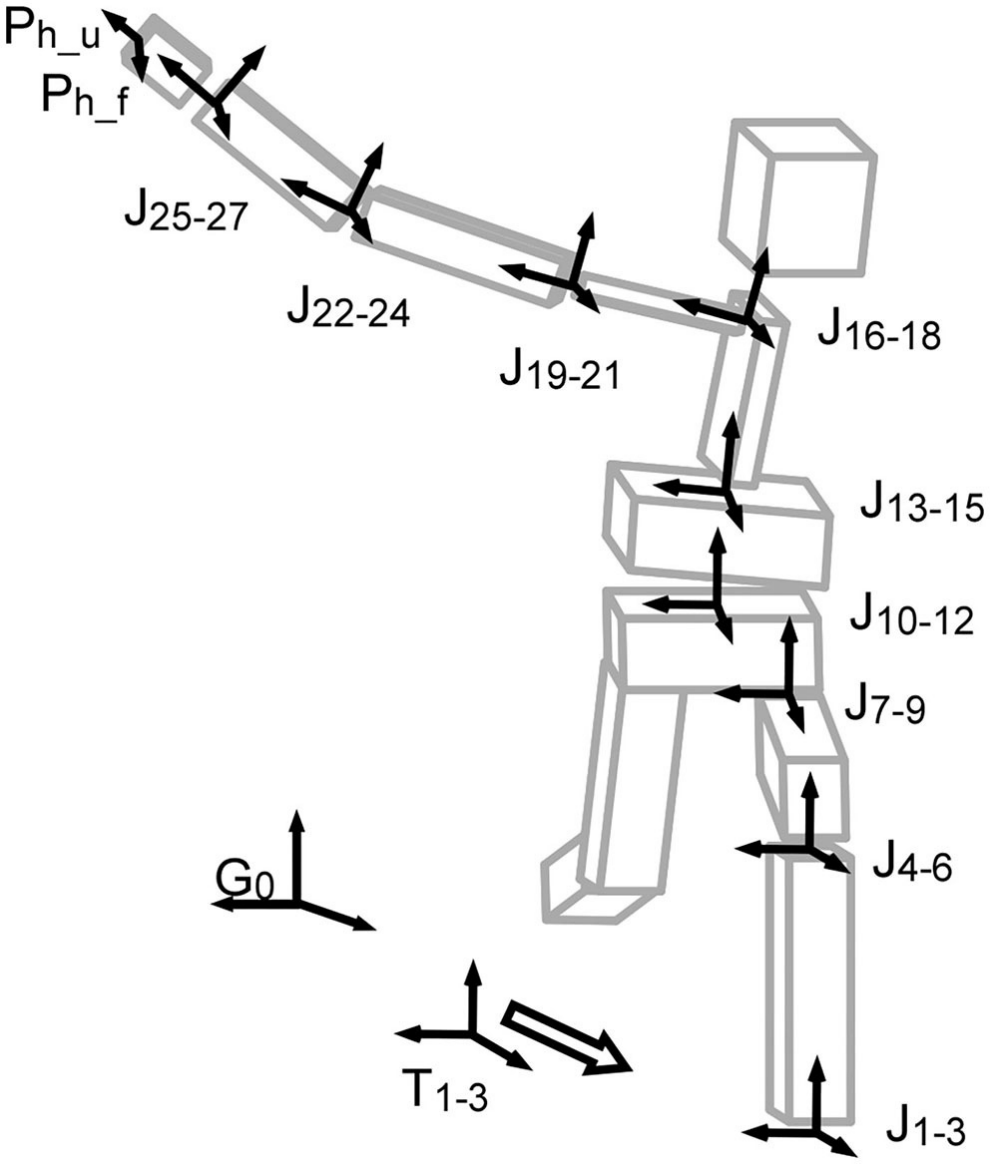
Multi-segment model and the global and local coordinate systems. P_h_ shows the hand vectors directing the distal end (P_h_u_), and the front of palm (P_h_f_). J_xx−yy_ shows a joint with three degrees of freedom from xx to yy. T_1−3_ shows the ankle translation with three degrees of freedom (x, y, and z components). G_0_ is the global coordinate system.

The 9-link-segment model used in this study was composed of shank and thigh of the stride leg, pelvis, abdomen, thorax, clavicle, and the upper arm, forearm, and hand of the throwing arm. Orthogonal coordinate systems were set for all local coordinate systems at each segment (Figure 1). Three DoFs of the angular component were set to each joint (ankle, knee, hip, low back (L5S1), middle of the trunk, sterno-clavicle, shoulder, elbow, and wrist), although the movement could be negligible for certain DoFs. Three DoFs were set to all joints to reduce calculation error in the direct kinematic with permutation method explained below.

In addition, three translational components were set to the distal end of the stride shank (ankle). In total, 30 DoFs were included in the direct kinematics model. Three angular components in the same joint were regarded as a set of DoFs in the permutation method because those components were the results calculated from one joint movement; see the details below (section Direct Kinematics With Permutation Method). Therefore, 30 DoFs were reduced to 10 DoFs (Supplementary Table 3).

The angles were calculated based on a conventional vector algebra and matrix method (Zatsiorsky, 1998), using the coordinate systems described in Figure 1 (see also Supplementary Table 3 and Supplementary Appendix 4).

### Direct Kinematics With Permutation Method

The permutation method was applied in the direct kinematics to test for covariation among the joint movements (Müller and Sternad, 2003). For the direct kinematics with the permutation method, a different motion from the measured one was reconstructed using different joint angle combination. Three DoFs of the measured joint angles in the same joint (for example, shoulder movements around three axes) were treated as one set of DoFs in the permutation method (Supplementary Table 3). Then ankle position and each joint movement were pseudo-randomly selected (permutated) among the corresponding DoF obtained in 10 successful trials for each participant, to artificially break off covariation in the measured joint-angle combination (covariation-free condition). The pseudo-random selection means the selection was based on permutation, i.e., selecting the corresponding DoF among the 10 successful trials randomly but not using the same trial twice for the other reconstructed motion. As a control condition, the measured angles themselves were selected and put into the direct kinematics calculation (measured condition) (Table 1).

**Table 1:** Reconstructed motion condition.

**Measured**	**Covariation-free**	**Measured-in-arm**	**Measured-in-leg**
**Wrist**	Wrist	**Wrist**	Wrist
**Elbow**	Elbow	**Elbow**	Elbow
**Shoulder**	Shoulder	**Shoulder**	Shoulder
**Sternoclavicle**	Sternoclavicle	**Sternoclavicle**	Sternoclavicle
**Thorax**	Thorax	Thorax	Thorax
**Pelvis**	Pelvis	Pelvis	Pelvis
**Hip**	Hip	Hip	**Hip**
**Knee**	Knee	Knee	**Knee**
**Ankle**	Ankle	Ankle	**Ankle**
**Ankle position**	Ankle position	Ankle position	**Ankle position**

*Joint angles and ankle position written in bold letters were used from the same trial, but those not written in bold letters were used from 10 pseudo-randomly selected successful trials for each participant except the beforementioned trial. Please see the text for details*.

To investigate which of the upper or lower extremities demonstrate the covariation affecting the variability of the result variables more effectively, the measured joint angle combination was used only for the corresponding DoF. The other joint angles were pseudo-randomly selected. Specifically, for the throwing arm, the measured joint angle combination was used for the sterno-clavicle, shoulder, elbow, and wrist joints (measured-in-arm condition). For the stride leg, the measured joint angle combination was used for the ankle position, the ankle joint angle, and the knee and hip joint angles (measured-in-leg condition). Ultimately, each participant had four different motion conditions (Table 1). The motion condition was used as the independent variable in subsequent statistical analyses.

One of merits of using this permutation method is that it is easy to understand the results intuitively because the mean and SD of each execution variable (joint angle) in any motion conditions are identical to those of the measured condition. Therefore, if variability in the result variables (the hand orientation and movement direction) increases in a reconstructed motion, it should result from decomposition in the specific combination (covariation free).

An alternative method, the uncontrolled manifold method, calculates the variability not contributing to the task-related variable as well as the variability contributing to the task-related variable. Since both variabilities are used for making an index, the index is affected by the variability not contributing to the variability of the result variable. In addition, it is difficult to set accurate values for the task-related variables in this study. Thus, the direct kinematics with the permutation method seemed to be preferable and adequate for the purpose of the current study.

### Dependent Variables

The trajectory of the pitched ball is solved by the simultaneous differential equations if the initial values are given. Here the initial values are the three-dimensional location of the ball, ball velocity, and the vector components of the rotational axis of the ball. The ball velocity can be expressed by the magnitude and direction. This direction means the movement direction of the ball. Since the ball is released from the hand, it can be assumed that the location and movement direction of the hand at the ball release are almost the same. The relationship between the movement direction of the hand is partly supported by the previous empirical study showing that the wrist movement direction around ball release correlated with the angle of the long axis of the ellipse representing the result of ball locations of 100 pitches (Shinya et al., 2017). Thus, the measures of hand movement direction (both the azimuth angle and elevation angle) were selected as a set of result variables.

In addition, it was reported that the intermediate and advanced table tennis players stabilized the orientation of the table tennis racket at ball impact to fulfill the task requirement, which was to hit the ball at a target on the table as quickly and accurately as possible (Iino et al., 2017). Even though the task is different from the ball throwing, the measures of hand orientation (instead of the racket) were selected as a second set of result variables.

Ten reconstructed motions for each condition were treated as a block to match with 10 measured motions for calculating mean and SD. Subsequently, 500 blocks were computed to bring the distribution closer to a normal distribution by eliminating the bias due to the pseudo-random selection for the covariation-free, measured-in-arm, and measured-in-leg conditions. The root mean square of SDs for the 500 blocks of the reconstructed motions was calculated and used as the dependent variable for each participant (Equation 1).

(1) $$\begin{array}{l} \sqrt{\frac{1}{N}\overset{500}{\underset{i=1}{\sum }}{\left(SD_{i}\right)}^{2}} \end{array}$$

Concretely speaking, SD of the azimuth of hand orientation at the instant of ball release (SD_azm_ang_), SD of the elevation of hand orientation at the instant of ball release (SD_elv_ang_), SD of the azimuth of hand movement direction at the instant of ball release (SD_azm_mov_), and SD of the elevation of hand movement direction at the instant of ball release (SD_elv_mov_) were used as the dependent variables.

**Figure 2:**
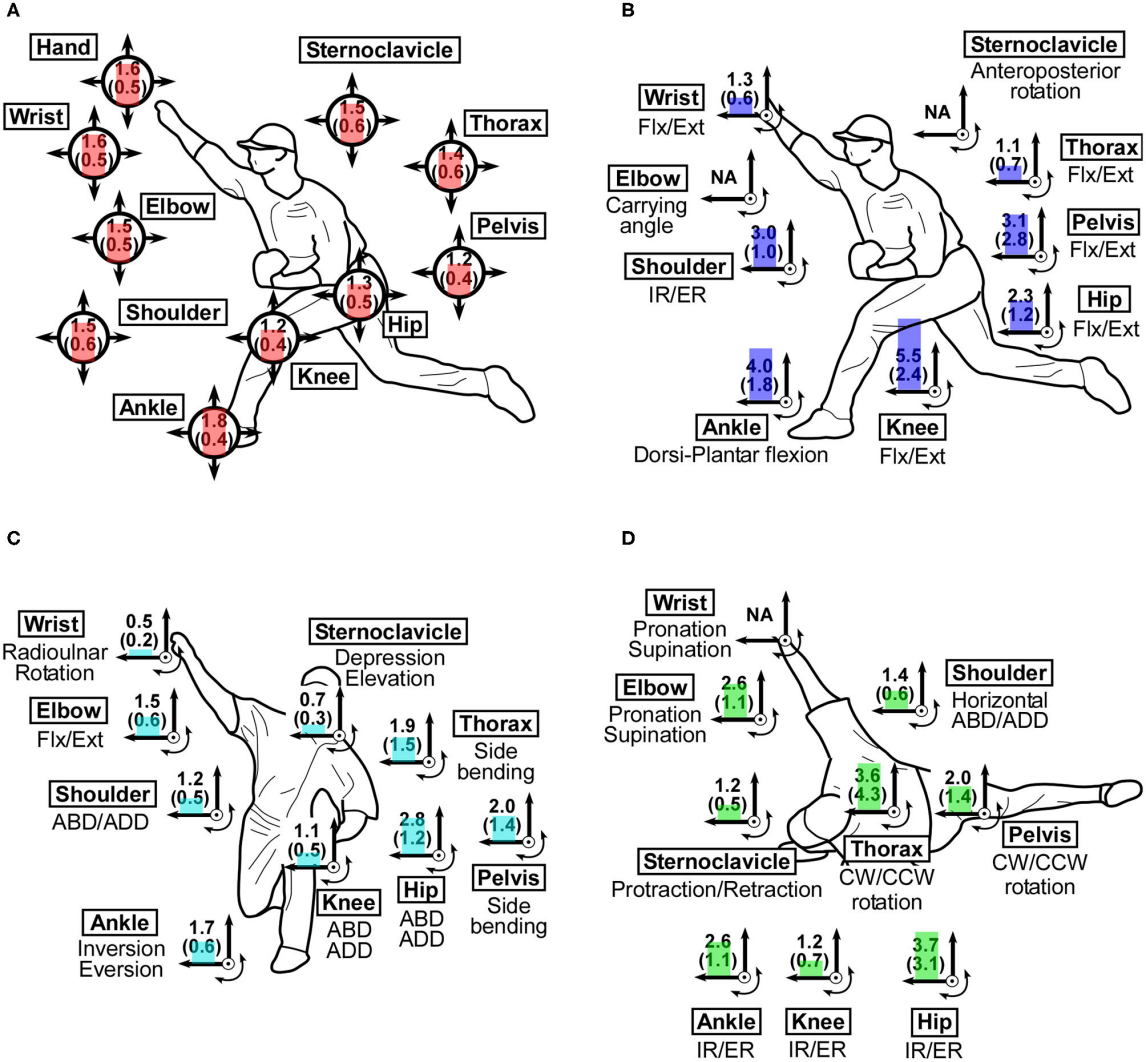
Standard deviations of joint angle at the instant of ball release in the successful trials in the measured condition. **(A)** SDs of joint position (cm). **(B)** SDs of joint angle in the sagittal plane (°). **(C)** SDs of joint angle in the frontal plane (°). **(D)** SDs of joint angle in the transverse plane (°). Flx/Ext, Flexion/extension; IR/ER, Internal/external rotation; ABD/ADD, Abduction/adduction; CW/CCW, Clockwise/counterclockwise rotation.

To grasp how joint movements varied among the measured trials, mean SDs of joint positions and joint angles in the measured condition were calculated (Figure 2).

### Statistics

Since some of the dependent variables among the participants violated the normality assumption, Friedman tests were conducted to compare the dependent variables among four conditions. To avoid inflation of type I error, Bonferroni correction was used to adjust probability values. Significance was set at 1.25%. When the main effects were revealed, Wilcoxon's *post-hoc* comparisons were performed with Bonferroni correction to compare every pair of the conditions. Notably, the significance level of the *post-hoc* test was set at 0.83%. SPSS statistics 26 (IBM Japan, Tokyo, Japan) was used for all statistical analyses.

## Results

### Variation of Joint Position and Angle in the Measured Trials

Mean SDs of joint position in the measured condition ranged from 0.012 to 0.018 m (Figure 2A). Mean SD of ankle position was largest and those 234 of knee position and pelvis position were smallest. Mean SDs of joint angles ranged from 0.5 to 5.5°. Among mean SDs of joint angle in the measured condition, the largest mean SD was found in flexion/extension movement in knee angle (5.5 ± 2.4°), followed by dorsi-plantar flexion in the ankle joint (4.0 ± 1.8°) (Figures 2B–D).

### Difference of the Dependent Variables Among Motion Conditions

**Figure 3:**
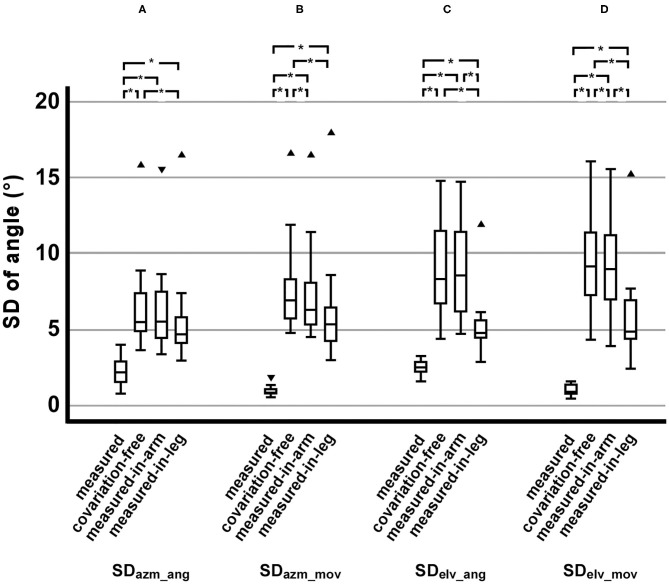
Comparisons of the dependent variables of the motion conditions. **(A)** SDs of the azimuth of hand orientation. **(B)** SDs of the azimuth of hand movement direction. **(C)** SDs of the elevation of hand orientation. **(D)** SDs of the elevation of hand movement direction. ▾: An outlier in the range of 1.5–3.0 box lengths from a hinge (interquartile range). ▴: An outlier beyond 3.0 box lengths from a hinge. *Significant difference (*p* < 0.0083, which corresponds to *p* < 0.05 with Bonferroni correction).

From the viewpoint of all dependent variables comprehensively, a similar trend in the difference of SDs among the motion conditions was observed. The SD in the measured condition was the smallest, followed by the measured-in-leg condition. The SD in the covariation-free condition was largest. The SD in the measured-in-arm condition was similar to the covariation-free (SD_azm_ang_ and SD_elv_ang_) condition or less but significantly smaller than the covariation-free (SD_azm_mov_ and SD_elv_mov_) condition. The SDs in the measured-in-leg condition were smaller than the measured-in-arm condition but larger than the measured condition (Figures 3A–D). Significant main effects of the motion conditions were observed in all dependent variables (for SD_pos_: χ^2^ = 32.5, *p* < 0.001; for SD_azm_ang_: χ^2^ = 27.3, *p* < 0.001; for SD_azm_mov_: χ^2^ = 31.3, *p* < 0.001; for SD_elv_ang_: χ^2^ = 31.3, *p* < 0.001; for SD_elv_mov_: χ^2^ = 34.0, *p* < 0.001).

#### Azimuth Angle of Hand Orientation

The SD_azm_ang_ in the measured condition was significantly smaller than those in all other conditions (*Z* = −3.06, *p* = 0.002, *r* = −0.884 for three conditions) (Figure 3A).

The SD_azm_ang_ in the measured-in-leg condition was significantly smaller than those in the covariation-free condition (*Z* = −3.06, *p* = 0.002, *r* = −0.884), while the SD_azm_ang_ in the measured-in-arm condition was not significantly smaller than that in the covariation-free condition (*Z* = −1.49, *p* = 0.136, *r* = −0.317) (Figure 3A). Mean SD_azm_ang_ in the measured-in-leg condition was smaller than that in the measured-in-arm condition, but it was not significant (*Z* = −2.118, *p* < 0.034, *r* = −0.611).

#### Azimuth Angle of Hand Movement Direction

The SD_azm_mov_ in the measured condition was significantly smaller than those in all other conditions (*Z* = −3.06, *p* = 0.002, *r* = −0.884 for three conditions) (Figure 3B).

Both SD_azm_mov_ in the measured-in-arm and measured-in-leg conditions were significantly smaller than those in the covariation-free condition (*Z* = −3.06, *p* = 0.002, *r* = −0.884 for the measured-in-arm condition and *Z* = −2.67, *p* = 0.008, *r* = −0.770 for the measured-in-leg condition) (Figure 3B). The SD_azm_mov_ in the measured-in-arm and measured-in-leg conditions were not significantly different (*Z* = −2.28, *p* = 0.023, *r* = −0.657) (Figure 3B).

#### Elevation Angle of Hand Orientation

The statistical difference pattern in the SD_elv_ang_ was similar to the SD_azm_ang_. The SD_elv_ang_ in the measured condition was significantly smaller than those in all other conditions (*Z* = −3.06, *p* = 0.002, *r* = −0.884 for the covariation-free and measured-in-arm conditions and *Z* = −2.98, *p* = 0.003, *r* = −0.861 for the measured-in-leg conditions) (Figure 3C).

The SD_elv_ang_ in the measured-in-leg condition was significantly smaller than those in the covariation-free condition and the measured-in-arm condition (*Z* = 3.06, *p* < 0.002, *r* = −0.884 for both conditions), while the SD_elv_ang_ in the measured-in-arm condition was not significantly smaller than that in the covariation-free condition (*Z* = −0.24, *p* = 0.814, *r* = −0.068) (Figure 3C).

#### Elevation Angle of Hand Movement Direction

Differences between all combinations were significant in the SD_elv_mov_. The SD_elv_mov_ in the measured condition was significantly smaller than those in all other conditions (*Z* = −3.06, *p* = 0.002, *r* = −0.884 for three conditions) (Figure 3D).

The SD_elv_mov_ in the measured-in-leg conditions were significantly smaller than those in the covariation-free and measured-in-arm conditions (*Z* = −3.06, *p* = 0.002, *r* = −0.884 for both conditions) (Figure 3D). The SD_elv_mov_ in the measured-in-arm was also significant smaller than that in the covariation-free condition (*Z* = −2.90, *p* = 0.004, *r* = −0.838) (Figure 3D).

## Discussion

Before discussing the differences in the dependent variables among the motion conditions, we want to mention the variability of the hand position in the measured condition to indicate how consistent the selected trials in the current study were.

The mean SD (±SD) of hand position was 0.016 ± 0.005 m. This SD was smaller than that accomplished by college pitchers recruited from an NCAA Division I baseball team, which was previously reported (0.057 ± 0.022 m in the horizontal axis and 0.038 ± 0.015 m in the vertical axis) (Whiteside et al., 2016). Since our result was calculated by the distance from the mean position in three-dimensional space, it is expected that the values would be smaller if converted to the same approach as used in the previous study. This suggested that the hand position at the instant of ball release among the successful trials for each participant was consistent.

### Covariation During Pitching

The first hypothesis regarding the utilization of covariation among the joint movements by skilled baseball pitchers to stabilize hand dynamics was supported completely. All statistics in the Z-values in the measured conditions, compared with those in the covariation-free condition, were −3.06, suggesting that the probability at which the measured condition belongs to the covariation-free condition is 0.37%. This means that the variability in each joint movement was not solely random noise, but it had some relationship with that in the other joint movement(s). The relationship might have contributed to reducing the variability in the hand dynamics. The fluctuation in joint movements, inevitably occurring among trials, seems to be controlled functionally.

These results agree with previous studies on different sports movements. The covariation in joint movements was observed between the elbow and the wrist joints during basketball free-throw shots (Button et al., 2003). A similar relationship was observed in the trunk and upper extremity during forehand drive in table tennis (Iino et al., 2017).

A previous study investigating the process of the practice of 1,300 flying-disc throws demonstrated that participants learned hand trajectory patterns in an earlier phase during practice, and that pattern of joint movements remained variable throughout the practice (Hung et al., 2008). It suggests that the stabilization of the hand trajectory (the result variable) was initially acquired to fulfill the task requirement; subsequently, exploring the optimal combination of joint movements (the execution variables) was maintained until the end of practice, to stabilize the hand trajectory. They concluded that skill acquisition consists of two learning processes: topology (intrinsic pattern of the end-point path) and dynamic control (represented by a joint coordination). The former is acquired early during practice and rapidly improves the task performance, while the latter occurs at a much slower rate and gradually improves the performance. The same two-stage processes have been proposed as adaptation and attunement (Whiting, 1984). The participants in the current study were expert baseball pitchers; therefore, they were regarded as being in the attunement stage. At least in the attunement stage, it appears that the covariation strategy, rather than the stochastic noise reduction strategy, takes responsibility for controlling the result variables. The question remains as to how the rate of contribution of the covariation strategy to the task performance changes as skill level changes. This needs to be clarified in future studies.

It was reported that an internal model was utilized for controlling the throwing arm and finger movements during throwing (Hore et al., 1999a,b). The internal model hypothesizes that there exists a model that emulates or simulates input/output characteristics of objects to be controlled. It includes both a forward model and an inverse-dynamic model. The forward model calculates trajectory of movement when some motor command is given as an input (called the efference copy), and the inverse-dynamic model calculates motor commands to achieve a desired trajectory of movement. With the feedback-error learning theory based on the concept of the internal model, the motor commands are generated based on the feedback of the error signal, which is calculated as the difference between actual joint movements and those calculated by the internal model. Simultaneously, the error signal is used in the inverse-dynamic model to generate the feed-forward motor commands. Both motor commands are combined as the final motor command to control the objects (Kawato et al., 1987). The internal model is updated by using the error signals in succession and is tuned so that the feed-forward motor commands execute the adequate joint movements for the task swiftly and accurately. However, the exact same movements cannot be repeated due to noise from various sources, even as learning progresses. The internal model always monitors errors by using the perception system, and this is reflected when controlling the upcoming movement. Therefore, perception and/or perception-action systems have an important role in this process.

Baseball pitching is a fast and quick movement, especially the duration of the arm acceleration phase, which is from the instant of maximum shoulder external rotation and ends at the instant of ball release, is only 0.028 ± 0.006 s (Fleisig et al., 1996). The error signal cannot be used in the ongoing movement but might be exploited in the upcoming movement. It implies that the internal model may be exploited to improve pitching performance. By tuning the internal model, the covariation might be strengthened, especially during the latter part of the process. Since the participants in the current study were expert pitchers, the covariation might have been strengthened and appeared as the significant difference of dependent variables between the measured condition and the covariation-free condition. However, this issue should be confirmed by future study employing less-experienced players.

### Upper Extremity vs. Lower Extremity

The second hypothesis was partly supported. All dependent variables in the measured-in-leg condition were significantly different from those in the covariation-free condition; however, only two of four dependent variables in the measured-in-arm condition were significantly different. Two of the four dependent variables in the measured-in-leg condition were significantly smaller than those in the measured-in-arm condition.

The length of the lever arm to the hand may partially account for the small SDs observed in the measured-in-leg condition compared to those in the measured-in-arm condition. However, the fact that the statistical significances were found only in the variables relating to the elevation angle but not the azimuth angle could not be accounted for solely by the long lever length. This could have resulted from the exclusive movement of the knee joint practically in the sagittal plane, suggesting that movement of the knee joint plays an important role in the covariation in joint movements for stabilizing the elevation angles of hand orientation and movement direction.

The other possible reason for the small SDs of the measured-in-leg condition is the difference in duration. The duration from stride foot contact to ball release is 0.145 ± 0.022 s, and that of the beginning of the arm acceleration phase (the maximum shoulder external rotation) to ball release is 0.028 ± 0.006 s (Fleisig et al., 1996). The time available for the upper extremity is too short for the sensory motor system to utilize the error signal. However, the time available for the lower extremity is longer than that for the upper extremity. Hand movement can be automatically modulated with short latencies (0.10–0.15 s) when the target to be reached unconsciously moves somewhat during the eye–hand coordination task (Prablanc and Martin, 1992; Saijo et al., 2005). Because the situation involving visuo-motor coordination to achieve the task is different from the situation involving sensory motor coordination for pitching, it is not clear whether automatic modulation can be utilized during the duration from stride foot contact to ball release. However, the longer duration may be an advantage because it may allow some information from the sensory system to be obtained.

Although the ankle and knee joint angles in the measured condition showed the largest variability, the knee and hip joint positions, which were determined by ankle and hip joint angle, showed the smallest variability. These results supported the theory that the covariation works largely in the stride leg. It was reported that the position of the center of mass was stabilized by utilizing the covariation in the leg during walking (Verrel et al., 2010; Monaco et al., 2018). Although the task did not involve walking in the current study, the covariation in the leg joints could have stabilized the center of mass or pelvis, resulting in the reduced variability of the hand trajectory. The properties of the pitcher's mound, such as slope, hardness, and condition of soil, are not always the same. Even if the mound is the same, the ground condition of the mound frequently changes due to use and weather conditions. Therefore, pitchers are required to be stable on an unstable mound. It seems that pitchers make their center of mass or pelvis stable using the covariation in the leg, to move the trunk and the throwing arm quickly. Thus, the covariation found in the stride leg may play a role in absorbing the unstable condition. It implies that regulating variabilities, rather than reducing variabilities, are necessary to pitch accurately.

### Limitations of the Study

Small sample size is one of the limitations of the study. It may have affected our ability to detect a few statistical differences of SD between the measured-in-arm and measured-in-leg conditions. However, some statistical differences were observed, and the results could support our hypotheses. The influence of the small sample size on the results was limited.

Using only the successful trials was another limitation. Ten successful trials were chosen in this study instead of all the trials. This means that our data were not fully representative of all fastball pitches. Therefore, the following possibilities remain. The participants might not always employ the covariation strategy, or the participants might employ the covariation strategy in the unsuccessful trials, too. However, the covariation strategy used in the successful trials provided beneficial information for baseball pitchers and for those who have concerns about baseball pitching.

In addition, the number of successful trials for calculating the SD is also one of the limitations. The task requirement that involved throwing a baseball at a target of 0.29 m in diameter, located ~20 m from the pitcher's plate, was similar to conditions in an actual competitive game. Several pitchers could not complete the task within 30 throws; however, some of the additional throws resulted from poor-quality motion capture, such as the removal of a marker. One pitcher made 44 throws. To maintain a high quality of pitching and to prevent throwing injuries in this study, the number of throws was not increased. Although SDs were calculated using 10 successful trials, SDs for reconstructed motions were calculated from 500 blocks. This helped minimize bias due to the pseudo-random selection.

The covariations in the throwing arm and the stride leg were investigated in this study in accordance with the practical perspective. The covariation found in the stride leg was greater than that in the throwing arm. However, the dependent variables in the measured-in-leg condition were significantly larger than those in the measured conditions, indicating that the reduction in the variability using the covariation in leg movements alone was insufficient to explain the minimal variability observed in the measured conditions. Different combinations may exist. A further detailed investigation is needed.

Covariation is caused by various aspects of movements, such as neural network reorganization, biarticular muscle function, and motion-dependent moment (Turvey et al., 1982). In this study, it could not be specified which aspect was primarily related to the covariation that occurred during pitching because the covariations in this study were considered only from the kinematic perspective. It may be necessary to deconstruct the covariations to understand the mechanism of the central nervous system that generates them; however, this was not possible during this study. Therefore, further investigation is required.

## Conclusion

The results imply that expert male baseball pitchers utilize covariations in joint movements to stabilize the hand movement at the instant of ball release and that the covariation in the stride leg is more important than that in the throwing arm for accurate and stable fastball pitching. Reducing the variability of each joint angle as well as utilizing the variability may be necessary for accurate and consistent fastball pitching.

## Data Availability Statement

The raw data supporting the conclusions of this article will be made available by the authors, without undue reservation.

## Ethics Statement

The studies involving human participants were reviewed and approved by the Institutional Research Ethics Committee at Osaka University. The patients/participants provided their written informed consent to participate in this study.

## Author Contributions

TM, TJ, and YM contributed the research design and data analysis to the study. TM, DH, DN, and YK contributed the data interpretation. TM wrote the first draft of the manuscript. All authors contributed to the data collection, manuscript revision, and final approval.

## Conflict of Interest

The authors declare that the research was conducted in the absence of any commercial or financial relationships that could be construed as a potential conflict of interest.
