# Supplementary material for: Consistent Hand Dynamics Are Achieved by Controlling Variabilities Among Joint Movements During Fastball Pitching
Source: Front Sports Act Living. 2020 Nov 17;2:579377. doi: 10.3389/fspor.2020.579377 (PMC7739665; doi:10.3389/fspor.2020.579377)
Supplement: Supplementary file 1 [file Presentation_1.pdf]

Appendix 4. Joint angle conventions

Matrix of direction cosines of *i* segment based on (*i-1*) segment:

$$M_{(i-1)i} = \begin{pmatrix} m_{(i-1)i\_11} & m_{(i-1)i\_12} & m_{(i-1)i\_13} \\ m_{(i-1)i\_21} & m_{(i-1)i\_22} & m_{(i-1)i\_23} \\ m_{(i-1)i\_31} & m_{(i-1)i\_32} & m_{(i-1)i\_33} \end{pmatrix} = \begin{pmatrix} \cos Xx & \cos Xy & \cos Xz \\ \cos Yx & \cos Yy & \cos Yz \\ \cos Zx & \cos Zy & \cos Zz \end{pmatrix},$$

$i = 1$: shank, $i = 2$: thigh, $i = 3$: pelvis, $i = 4$: abdomen, $i = 5$: thorax, $i = 6$: clavicle, $i = 7$: upper arm, $i = 8$: forearm, $i = 9$: hand

(*X, Y, Z*): unit vectors composing the local reference system of the (*i-1)* segment.

$(x, y, z)$: unit vectors composing the local reference system of the *i* segment.

Rotation matrix:

$$R_{jx} = \begin{pmatrix} 1 & 0 & 0 \\ 0 & \cos\alpha_j & -\sin\alpha_j \\ 0 & \sin\alpha_j & \cos\alpha_j \end{pmatrix},$$

$$R_{jy} = \begin{pmatrix} \cos\beta_j & 0 & \sin\beta_j \\ 0 & 1 & 0 \\ -\sin\beta_j & 0 & \cos\beta_j \end{pmatrix},$$

$$R_{jz} = \begin{pmatrix} \cos\gamma_j & -\sin\gamma_j & 0 \\ \sin\gamma_j & \cos\gamma_j & 0 \\ 0 & 0 & 1 \end{pmatrix}$$

$$R_j = R_{jx}R_{jy}R_{jz}$$

$$R_j = \begin{pmatrix} \cos\beta_j\cos\gamma_j & -\cos\beta_j\sin\gamma_j & \sin\beta_j \\ \cos\alpha_j\sin\gamma_j + \sin\alpha_j\sin\beta_j\cos\gamma_j & \cos\alpha_j\cos\gamma_j - \sin\alpha_j\sin\beta_j\sin\gamma_j & -\sin\alpha_j\cos\beta_j \\ \sin\alpha_j\sin\gamma_j - \cos\alpha_j\sin\beta_j\cos\gamma_j & \sin\alpha_j\cos\gamma_j + \cos\alpha_j\sin\beta_j\sin\gamma_j & \cos\alpha_j\cos\beta_j \end{pmatrix}$$

$j = 1$: ankle, $j = 2$: knee, $j = 3$: hip, $j = 4$: low back, $j = 5$: middle of trunk, $j = 6$: sternoclavicle, $j = 7$: shoulder, $j = 8$: elbow, $j = 9$: wrist

Joint *j* angles (x-axis): $\alpha_{j\_x} = \tan^{-1}\left(-\frac{m_{(i-1)i\_23}}{m_{(i-1)i\_33}}\right)$

Joint *j* angles (y-axis): $\beta_{j\_y} = \sin^{-1}\left(m_{(i-1)i\_13}\right)$ or $\tan^{-1}\left(\frac{m_{(i-1)i\_13}}{\sqrt{m_{(i-1)i\_11}{}^2 + m_{(i-1)i\_12}{}^2}}\right)$

Joint *j* angles (z-axis): $\gamma_{j\_z} = \tan^{-1}\left(-\frac{m_{(i-1)i\_12}}{m_{(i-1)i\_11}}\right)$
