# Supplementary material for: Consistent Hand Dynamics Are Achieved by Controlling Variabilities Among Joint Movements During Fastball Pitching
Source: Front Sports Act Living. 2020 Nov 17;2:579377. doi: 10.3389/fspor.2020.579377 (PMC7739665; doi:10.3389/fspor.2020.579377)
Supplement: Supplementary file 2 [file Table_1.pdf]

Appendix 1

Supplementary Table 1. Locations of the retro-reflective markers on the body.

Head	Head	Vertex
	Front head	Forehead along the anterior median line
	Back head	External occipital protuberance along with the posterior median line
Torso	Manubrium	Manubriosternal edge
	Xiphoid	Superior edge of the xiphoid process
	C7	Seventh cervical vertebra
	T8	Eight thoracic vertebrae
	Scapula	Scapula for asymmetry identification
	ASIS	Anterior superior iliac spine (L/R)
	PSIS	Posterior superior iliac spine (L/R)
Leg & Foot	Thigh	Arbitrary position between the hip and knee (L/R)
	Lateral knee	Lateral epicondyle of the femur (L/R)
	Medial knee	Medial epicondyle of the femur (L/R)
	Shank	Arbitrary position between knee and ankle (L/R)
	Lateral ankle	Lateral malleolus (L/R)
	Medial ankle	Medial malleolus (L/R)
	Toe	Second metatarsophalangeal joint (L/R)
	Heel	Calcaneus at the same height above the plantar surface as TOE (L/R)
	5th MCP joint	Fifth metacarpal head (throwing side)
Arm	Shoulder	Acromial edge (L/R)
	Anterior Shoulder	Lesser tubercle of the humerus (throwing side)
	Posterior Shoulder	Posterior side of the above marker (throwing side)
	Upper arm	Arbitrary position between the shoulder and elbow (L/R)
	Lateral elbow	Lateral epicondyle of the humerus (L/R)
	Medial elbow	Medial epicondyle of the humerus (L/R)
	Forearm	Arbitrary position between the elbow and wrist (L/R)
	Lateral wrist	Radial styloid process (L/R)
	Medial wrist	Ulnar styloid process (L/R)
Hand & Ball	3rd MCP joint	Third metacarpal head (L/R)
	(PIP joint)	Distal end of the third proximal phalange of the throwing side
	(DIP joint)	Distal end of the third distal phalange of the throwing side
	Tip of finger	Center of nail of the third finger of the throwing side
	Ball 1-4	Arbitrary position not to disturb pitching

Note: 50 markers were placed on the body and 4 markers on the ball. The diameter of markers on the finger and ball was 0.006 m and that of the others was 0.014 m. Two markers on the 3rd finger (PIP joint and DIP joint) were placed for the other study with a different purpose.
