# Supplementary material for: Consistent Hand Dynamics Are Achieved by Controlling Variabilities Among Joint Movements During Fastball Pitching
Source: Front Sports Act Living. 2020 Nov 17;2:579377. doi: 10.3389/fspor.2020.579377 (PMC7739665; doi:10.3389/fspor.2020.579377)
Supplement: Supplementary file 3 [file Table_2.pdf]

Appendix 2

Supplementary Table A2. Ball velocity (m/s) and ball radial error (deg). Part 1

Pitcher	ID		IR		IS		IY		NS		OS	
Trial#	VEL	DIS	VEL	DIS	VEL	DIS	VEL	DIS	VEL	DIS	VEL	DIS
1	38.6	689	33.1	223	35.0	654	37.8	364	36.4	313	37.2	309
2	38.6	254	33.2	413	35.0	225	38.3	80	38.3	143	36.7	63
3	38.1	158	32.0	459	35.6	222	38.1	291	36.1	421	35.3	230
4	38.1	773	32.5	225	34.6	122	38.1	284	35.8	409	36.4	256
5	38.1	267	32.0	231	—	245	38.3	437	36.9	427	36.4	553
6	37.8	158	36.4	469	33.9	76	37.5	267	38.3	215	36.4	426
7	37.8	382	37.1	63	34.2	174	37.8	38	38.1	285	36.9	160
8	37.8	88	36.8	391	34.2	650	38.3	73	37.2	61	36.7	331
9	37.2	95	36.6	581	—	117	37.8	27	36.7	392	36.4	404
10	37.8	379	35.6	310	34.7	407	38.1	381	35.8	83	36.1	93
11	36.4	153	36.3	628	—	291	38.3	90	36.1	108	36.7	220
12	36.9	193	35.3	71	36.7	124	38.3	162	36.1	432	37.5	80
13	36.9	371	35.7	364	36.4	189	38.3	424	35.6	530	36.7	279
14	36.4	167	35.8	567	37.2	13	38.3	191	37.8	361	36.9	81
15	36.4	131	35.6	169	37.5	198	38.3	284	36.7	118	36.7	415
16	36.9	484	34.7	431	34.4	671	38.3	151	36.7	199	34.2	183
17	35.8	105	34.9	402	34.4	36	37.8	230	36.7	435	34.7	384
18	36.1	534	35.7	204	35.3	342	38.1	161	37.5	227	—	589
19	36.4	18	34.2	135	33.6	250	38.3	47	36.7	261	33.9	147
20	37.8	326	35.4	101	34.7	526	38.3	239	37.5	79	33.6	243
21	38.1	264	34.2	10	34.7	263	38.6	150	36.7	395	37.5	334
22	37.8	101	33.3	223	36.1	374	38.3	560	36.9	65	37.5	406
23	38.9	96	32.6	529	—	319	38.6	89	36.1	281	37.2	562
24	38.6	186	32.8	214	36.4	233	38.6	543	36.1	266	36.4	253
25	38.1	373	32.5	118	35.8	137	38.3	462	36.9	269	37.2	178
26	37.2	95	36.1	58	34.7	306	38.1	245	36.7	147	38.3	215
27	37.2	332	36.2	220	34.2	566	37.8	84	36.4	273	37.8	366
28	38.1	419	36.1	815	35.6	137	37.5	111	36.9	186	37.5	269
29	36.9	237	35.6	551	34.4	466	37.8	288	36.7	257	38.9	287
30	38.1	177	36.1	554	35.3	382	37.8	381	37.8	177	39.2	186
Mean	37.5	267	34.8	324	35.2	291	38.1	238	36.8	260	36.6	283
SD	0.8	181	1.6	204	1.0	183	0.3	152	0.7	130	1.3	141

VEL: ball velocity (m/s). DIS: ball radial error (mm). Trials surrounded by square were selected to analyze as "successful trials."

Supplementary Table A2. Ball velocity (m/s) and ball radial error (deg). Part 2

Pitcher	TU		YO		YZ		IW				
Trial#	VEL	DIS	VEL	DIS	VEL	DIS	VEL	DIS	No.	VEL	DIS
1	36.1	543	36.1	397	36.4	448	35.3	463	31	33.9	523
2	36.7	226	36.9	436	36.9	553	36.1	660	32	33.9	352
3	37.2	334	37.8	572	36.9	476	36.7	467	33	36.4	376
4	37.2	393	36.9	241	37.2	249	36.1	299	34	34.2	713
5	37.5	189	37.8	160	37.2	168	35.6	140	35	35.6	229
6	36.9	108	38.1	497	37.5	237	35.8	421	36	35.6	725
7	36.9	90	38.1	177	37.5	181	35.6	411	37	35.3	372
8	37.5	335	38.6	414	36.7	612	35.8	185	38	35.6	439
9	37.8	338	37.8	170	36.9	453	34.4	327	39	34.4	580
10	37.5	336	37.2	402	36.9	161	35.0	318	40	36.1	103
11	38.6	129	38.1	316	34.2	714	37.5	181	41	36.9	463
12	38.3	194	37.2	267	36.1	150	37.8	104	42	36.4	125
13	39.2	542	37.8	81	35.8	28	36.7	313	43	35.3	370
14	38.9	116	37.8	319	35.6	337	35.8	493	44	35.8	97
15	38.9	476	38.6	90	35.8	466	37.5	210	–	–	–
16	35.0	409	36.4	587	36.9	334	34.2	320	–	–	–
17	36.4	286	38.1	321	36.4	166	33.3	438	–	–	–
18	36.7	430	38.1	204	36.7	220	34.2	219	–	–	–
19	37.2	201	38.6	137	36.1	252	34.4	250	–	–	–
20	37.2	217	38.6	399	36.9	197	34.2	88	–	–	–
21	37.5	217	38.1	604	36.4	310	37.5	411	–	–	–
22	37.5	411	38.6	204	35.6	145	37.5	492	–	–	–
23	37.5	121	38.6	352	35.6	201	35.3	246	–	–	–
24	37.8	29	38.6	162	35.8	290	36.7	263	–	–	–
25	37.8	186	38.9	520	35.3	172	35.0	726	–	–	–
26	38.9	421	37.2	589	37.2	500	35.6	693	–	–	–
27	38.9	33	38.1	480	37.2	330	35.0	187	–	–	–
28	39.2	558	38.1	171	37.2	188	35.0	240	–	–	–
29	38.6	140	38.1	63	37.2	180	35.6	150	–	–	–
30	39.4	204	38.1	155	36.9	454	35.6	446	–	–	–
Mean	37.7	274	37.9	316	36.5	306	–	–		35.6	355
SD	1.0	154	0.7	168	0.8	163	–	–		1.1	180

VEL: ball velocity (m/s). DIS: ball radial error (mm). Trials surrounded by square were selected to analyze as "successful trials."

Supplementary Table A2. Ball velocity (m/s) and ball radial error (deg). Part 3

Pitcher	NB					SR				
Trial#	VEL	DIS	No	VEL	DIS	VEL	DIS	No	VEL	DIS
1	34.8	545	31	35.3	370	36.7	639	31	35.3	857
2	35.5	368	32	35.0	336	37.1	208	32	35.0	321
3	35.7	156	33	35.6	109	37.4	310	33	35.4	203
4	35.1	149	34	35.3	266	37.2	480	34	35.0	156
5	35.8	94	35	35.0	121	37.7	266	35	35.4	316
6	36.4	729	–	–	–	36.1	122	36	35.0	362
7	37.0	233	–	–	–	36.3	127	37	34.3	255
8	36.9	431	–	–	–	37.1	250	38	34.4	471
9	37.2	316	–	–	–	36.4	367	39	34.6	43
10	36.3	602	–	–	–	36.3	21			
11	36.2	117	–	–	–	37.8	459			
12	37.0	269	–	–	–	36.9	536			
13	35.5	602	–	–	–	37.2	559			
14	36.3	61	–	–	–	36.6	200			
15	36.4	245	–	–	–	37.0	170			
16	36.7	363	–	–	–	36.9	514			
17	36.9	261	–	–	–	36.5	252			
18	36.4	262	–	–	–	36.8	258			
19	36.9	243	–	–	–	36.5	396			
20	36.4	350	–	–	–	37.1	155			
21	37.8	393	–	–	–	36.2	407			
22	36.7	452	–	–	–	36.8	275			
23	37.8	316	–	–	–	35.0	314			
24	38.1	81	–	–	–	35.8	88			
25	37.8	186	–	–	–	36.8	107			
26	35.0	255	–	–	–	37.1	298			
27	34.4	468	–	–	–	36.1	144			
28	34.4	270	–	–	–	36.5	218			
29	35.0	345	–	–	–	36.3	329			
30	35.0	196	–	–	–	35.6	312			
Mean	–	–		36.1	302	–	–		36.3	302
SD	–	–		1.0	159	–	–		0.9	172

VEL: ball velocity (m/s). DIS: ball radial error (mm). Trials surrounded by square were selected to analyze as "successful trials."
