# Supplementary material for: Consistent Hand Dynamics Are Achieved by Controlling Variabilities Among Joint Movements During Fastball Pitching
Source: Front Sports Act Living. 2020 Nov 17;2:579377. doi: 10.3389/fspor.2020.579377 (PMC7739665; doi:10.3389/fspor.2020.579377)
Supplement: Supplementary file 4 [file Table_3.pdf]

Appendix 3

Supplementary Table A3. Degrees of freedom and joint angle conventions

30 DOF	Set of DOF	Joint	Axes	Direction of movement
T_1	1	Stride ankle position	x	(+): rightward, (-): leftward
T_2			y	(+): forward, (-): backward
T_3			z	(+): upward, (-): downward
J_1	2	Stride ankle	x	0 deg=neutral, (+): plantar flexion, (-): dorsiflexion
J_2			y	0 deg=neutral, (+): supination, (-): pronation
J_3			z	0 deg=neutral, (+): adduction, (-): abduction
J_4	3	Stride knee	x	0 deg=neutral, (+): flexion, (-): hyperextension
J_5			y	0 deg=neutral, (+): adduction, (-): abduction
J_6			z	0 deg=neutral, (+): internal rotation, (-): external rotation
J_7	4	Stride hip	x	0 deg=neutral, (+): hyperextension, (-): flexion
J_8			y	0 deg=neutral, (+): adduction, (-): abduction
J_9			z	0 deg=neutral, (+): internal rotation, (-): external rotation
J_{10}	5	Low back	x	0 deg=neutral, (+): hyperextension, (-): flexion
J_{11}			y	0 deg=neutral, (+): right bending, (-): left bending
J_{12}			z	0 deg=neutral, (+): abdomen CCW rotation, (-): abdomen CW rotation
J_{13}	6	Middle of trunk	x	0 deg=neutral, (+): hyperextension, (-): flexion
J_{14}			y	0 deg=neutral, (+): right bending, (-): left bending
J_{15}			z	0 deg=neutral, (+): trunk CCW rotation, (-): trunk CW rotation
J_{16}	7	Sterno-clavicle	x	0 deg=neutral, (+): posterior rotation, (-): anterior rotation
J_{17}			y	0 deg=horizontal, (+): depression, (-): elevation
J_{18}			z	0 deg=parallel to the shoulders, (+): protraction, (-): retraction
J_{19}	8	Throwing shoulder	x	0 deg=90 deg Abd & Ext, (+): external rotation, (-): internal rotation
J_{20}			y	0 deg=90 deg Abd, (+): adduction, (-): abduction
J_{21}			z	0 deg=neutral, (+): horizontal adduction, (-): horizontal abduction
J_{22}	9	Throwing elbow	x	0 deg=neutral, (+): valgus rotation, (-): varus rotation
J_{23}			y	0 deg=90 deg flexion, (+): extension, (-): flexion
J_{24}			z	0 deg=90 deg supination, (+): supination, (-): pronation
J_{25}	10	Throwing wrist	x	0 deg=neutral, (+): radial rotation, (-): ulnar rotation
J_{26}			y	0 deg=neutral, (+): extension, (-): flexion
J_{27}			z	0 deg=neutral, (+): supination, (-): pronation

CW: clockwise, CCW: counter clockwise, Abd: abduction, Ext: external rotation
